# *Bacillus subtilis* SL18r Induces Tomato Resistance Against *Botrytis cinerea*, Involving Activation of Long Non-coding RNA, MSTRG18363, to Decoy miR1918

**DOI:** 10.3389/fpls.2020.634819

**Published:** 2021-02-03

**Authors:** Cheng Zhou, Jingjing Zhu, Nana Qian, Jiansheng Guo, Congsheng Yan

**Affiliations:** ^1^Jiangsu Provincial Key Lab of Solid Organic Waste Utilization, Jiangsu Collaborative Innovation Center of Solid Organic Wastes, Educational Ministry Engineering Center of Resource-Saving Fertilizers, Nanjing Agricultural University, Nanjing, China; ^2^Key Lab of Bio-Organic Fertilizer Creation, Ministry of Agriculture and Rural Affairs, Anhui Science and Technology University, Bengbu, China; ^3^Institute of Horticulture, Anhui Academy of Agricultural Sciences, Hefei, China; ^4^School of Medicine, Zhejiang University, Hangzhou, China

**Keywords:** beneficial rhizobacteria, induced systemic resistance, long non-coding RNA, *Botrytis cinerea*, comparative transcriptome

## Abstract

Mounting evidence has indicated that beneficial rhizobacteria can suppress foliar pathogen invasion via elicitation of induced systemic resistance (ISR). However, it remains elusive whether long non-coding RNAs (lncRNAs) are involved in the mediation of the rhizobacteria-primed ISR processes in plants. Herein, we demonstrated the ability of the rhizobacterial strain *Bacillus subtilis* SL18r to trigger ISR in tomato plants against the foliar pathogen *Botrytis cinerea*. Comparative transcriptome analysis was conducted to screen differentially expressed lncRNAs (DELs) between the non-inoculated and SL18r-inoculated plants. Among these DELs, four variants of MSTRG18363 possessed conserved binding sites for miR1918, which negatively regulates immune systems in tomato plants. The expression of MSTRG18363 in tomato leaves was significantly induced by SL18r inoculation. The transcription of MSTRG18363 was negatively correlated with the expression of miR1918, but displayed a positive correlation with the transcription of the RING-H2 finger gene *SlATL20* (a target gene of miR1918). Moreover, MSTRG18363-overexpressing plants exhibited the enhanced disease resistance, reduction of miR1918 transcripts, and marked increases of *SlATL20* expression. However, the SL18r-induced disease resistance was largely impaired in the MSTRG18363-silenced plants. VIGS-mediated *SlATL20* silencing also greatly weakened the SL18r-induced disease resistance. Collectively, our results suggested that induction of MSTRG18363 expression in tomato plants by SL18r was conducive to promoting the decoy of miR1918 and regulating the expression of *SlATL20*, thereby provoking the ISR responses against foliar pathogen infection.

## Introduction

In plants, a series of intricate strategies has been evolved to defend against pathogenic bacteria, fungi and virus. Besides physical and chemical defense, plants develop sophisticatedly inducible immune systems that can be quickly evoked by pathogen-derived elicitors. The inducible defense responses are tightly controlled by several hormone such as salicylic acid (SA) and jasmonic acid (JA) (De Vos et al., 2006; Pozo and Azcón-Aguilar, 2007; Asselbergh et al., 2008). Apart from basal immune systems that can be stimulated at the pathogen-infected sites, a primed defense strategy has also been developed by plants, in which defense responses have not been activated for increasing plant’s resistance, but is attributable to quicker induction of defense-related signaling pathways upon exposure to the attacks of pathogens (Bostock, 2005). In contrast to constitutive defense, initiation of priming defense exhibits less fitness costs of disease resistance in plants (van Hulten et al., 2006).

Well-studied examples of inducible defenses in plants are the activated systemic acquired resistance (SAR) by necrosis-inducing pathogen infection (Grant and Lamb, 2006). Initiation of SAR is required for locally and systemically increasing SA synthesis in plants, which result in transcriptional changes of a variety of pathogenesis-related (PR) proteins (Maleck et al., 2000; Molinari et al., 2014). The regulatory protein NPR1 is essential for transducing the SA signals in *Arabidopsis* (Ding et al., 2018). Overexpression of the *nahG* gene encoding a putative salicylate hydroxylase (SA-degrading enzyme) in transgenic plants fails to initiate SAR and activating the transcription of PR-related genes (Yang et al., 2004). The other example of the induced plant resistance is inducible systemic resistance (ISR), which can be effectively elicited by beneficial rhizobacteria strains (Lugtenberg and Kamilova, 2009). In contrast to SAR, initiation of the ISR responses is closely related to the stimulation of the JA- and ET-dependent pathways, involving upregulation of the expression of defensin 1.2 (*PDF1.2*) (De Vleesschauwer et al., 2008; Takishita et al., 2018). However, the SA-related signaling pathways can be triggered in plants exposed to several PGPR strains (Yan et al., 2002; Ryu et al., 2003). Rhizobacteria strain *B. subtilis* FB17 has been reported to activate both the SA- and ABA-related pathways to prevent the stomata-mediated entering routes of foliar pathogen into *Arabidopsis* plants (Kumar et al., 2012). *Bacillus cereus* AR156 can provoke ISR of *Arabidopsis* plants against pathogenic bacteria via stimulation of the JA-/ET- and SA-related pathways (Nie et al., 2017).

In nature, plant roots can recognize a myriad of microbe-derived elicitors for successful establishment of ISR. Bacteria-derived signals such as lipopolysaccharides, volatile organic compounds (VOCs), siderophores and cyclic lipopeptides have been identified to stimulate ISR responses in plants (Ongena et al., 2007; Aznar and Dellagi, 2015; Tahir et al., 2017). For example, the VOCs released by rhizobacterial strain *B. subtilis* GB03 can trigger the ISR responses in *Arabidopsis* plants via the ET-dependent pathways (Sharifi and Ryu, 2016). Cyclic lipopeptides from *B. subtilis* can provoke ISR by triggering the SA- and JA-dependent pathways (Ongena et al., 2007). MicroRNAs (miRNAs) have recently been demonstrated to participate in the mediation of rhizobacteria-mediated ISR processes in plants. *B. cereus* AR156 can effectively provoke the ISR responses in *Arabidopsis* plants against pathogenic bacteria via repression of miR825 and miR825^∗^ (Niu et al., 2016). *Bacillus amyloliquefaciens* FZB42 inhibits the expression of miR846 to elicit the ISR responses via the JA-related pathway (Xie et al., 2018). It is increasingly evidenced that long non-coding RNAs (lncRNAs) can mediate plant defense responses against pathogen infection by decoying miRNAs. In tomato plants, Slylnc0195 regulates the expression of class III HD-Zip TF genes by decoying miR166 in the yellow leaf curl virus-infected tomato plants (Wang et al., 2015). LncRNA39026 induces tomato resistance against *Phytophthora infestans* via promoting the decoy of miR168a and the transcription of PR-related genes (Hou et al., 2020). LncRNA23468 modulate the expression of NBS-LRR genes in the *P. infestans*-infected tomato by decoying miR482b (Jing et al., 2019). Despite the critical roles of lncRNAs in plant immunity, the functions of lncRNAs in the rhizobacteria-induced ISR remain sparsely explored.

Aiming at dissecting the ISR responses in tomato plants, we explored the mechanisms underpinning beneficial rhizobacteria *Bacillus subtilis* SL18r elicited ISR at the lncRNA level. To identify tomato lncRNAs that mediated the ISR responses provoked by *B. subtilis* SL18r, we sequenced lncRNA species by high-throughput sequencing and found that the transcription levels of MSTRG18363 were evidently induced in tomato leaves by root inoculation with *B. subtilis* SL18r. Upon exposure to *Botrytis cinerea*, the SL18r-inoculated plants had higher levels of MSTRG18363 expression than the control plants. Compared with the wild-type (WT) plants, the MSTRG18363-overexpressing plants exhibited the enhanced resistance of transgenic tomato plants to *B. cinerea*. However, virus-mediated silencing of MSTRG18363 largely weakened the SL18r-induced resistance against the invasion of *B. cinerea*. Furthermore, we further demonstrated that overexpression of MSTRG18363 in transgenic tomato plants markedly suppressed the expression of miR1918, a negative regulator of plant immunity. These findings indicated that the MSTRG18363-mediated decoy of miR1918 contributed to the SL18r-induced ISR in tomato plants.

## Materials and Methods

### Bacterial Inoculation and Pathogen Infection Assays

*Bacillus subtilis* SL18r isolated from tomato rhizospheric soils was identified by sequencing of 16S rRNA genes (GenBank No. MW269558). *B. subtilis* SL18r was cultured for 16 h at 30°C and then centrifuged at 8,000 × *g*, followed by resuspension in sterile 0.05 M phosphate buffered saline (PBS) solution (pH 7.2). Finally, sterilized (autoclaved) soil was poured with cell suspensions of SL18r at the indicated concentrations, and the sterilized (autoclaved) soil treated with the PBS solution were used as the control. For preparing the inoculum of *Botrytis cinerea*, the fungal pathogen was cultured for 2 weeks and spore suspensions were then prepared with the solution containing 0.01 M KH_2_PO_4_ and 6.67 mM glucose to a density of 2 × 10^5^ spores ml^–1^ (Curvers et al., 2010).

After 3 days (d) of bacterial inoculation, tomato plants were used to perform *in vivo* and *in vitro* assays of pathogen inoculation as reported by El Oirdi et al. (2011) with minor modifications: *in vitro* tests (pathogenicity tests on detached leaves), in which 5 μl droplet of spore suspension was inoculated on one side of the midvein of detached leaves and incubated in moist petri dishes at 23°C and *in vivo* tests (whole-plant inoculation), in which the uniform plants were selected and sprayed with spore suspension, and then cultivated at 23°C with 90% relative humidity (14 h day/10 h night cycle). At 5 days post infection (dpi), lesion diameters of detached leaves and disease index were measured as described recently by Jing et al. (2019). In addition, the transcription of *B. cinerea* actin gene (*BcActin*) was quantified for measuring the biomass of *B. cinerea* in leaves, and the tomato actin gene (*SlActin*) was used as a control as reported recently by Hu et al. (2018). The ratio of *BcActin* to *SlActin* was used to evaluate relative biomass of *B. cinerea*.

### RNA-Sequencing (RNA-Seq)

To perform RNA-Seq, 7-day-old tomato seedlings were initially cultivated in soil for 3 weeks and were then poured with cell suspension of SL18r at a final density of 1 × 10^8^ CFU g^–1^ soil for 3 days, and tomato plants poured with the PBS solution were used as the control. Subsequently, the control and inoculated leaves were sprayed with 2 × 10^5^ spore ml^–1^ spore suspension of *B. cinerea*. At 0, 24, and 48 h post infection, the control and inoculated leaves were used to construct the RNA-Seq libraries. Total RNAs were isolated from leaf samples using the TRIzol reagents (Invitrogen, United States). The obtained RNA samples were applied to construct RNA-Seq libraries through the Illumina Hiseq 4000 platform. Raw sequencing data were submitted to the NCBI Sequence Read Archive (SRA) database (No. PRJNA679081). Finally, the clean reads were mapped to a reference tomato genome iTAGv2.3 as described previously by Trapnell et al. (2009). Comparative analyses between the RNA-Seq libraries of the control and inoculated plants were applied to identify differentially expressed lncRNAs (DELs). The expression of lncRNAs was determined by the normalized read counts using Cufflinks. The DELs were identified using R package DEGseq with log_2_ (fold change) ≥1.0 and a false discovery rate (FDR) adjusted *P* ≤ 0.05 (Anders and Huber, 2010).

### Construction of MSTRG18363-, Mutated MSTRG18363-, and miR1918-Overexpressing Plasmids

The sequences of four MSTRG18363 variants were obtained by RNA-Seq in this study (Supplementary Figure 1). The MSTRG18363 variants were amplified by PCR, and positive clones were sequenced. The MSTRG18363 sequences were digested and ligated into the plant binary vector pCAMBIA1300. An artificially synthesized form of MSTRG18363 (asf-MSTRG18363, consensus sequence of four MSTRG18363 variants; Supplementary Figure 2) was also inserted into the plant binary vector pCAMBIA1300. Furthermore, a mutated lncRNA23468 carrying six base mutation paring with miR1918 (masf-MSTRG18363; Supplementary Figure 3) was artificially synthesized by Tsingke Biotech (Nanjing, China) and cloned into the pCAMBIA1300. Moreover, genomic DNA from tomato plants was used as the template to amplify the precursor sequence of miR1918 (miRBase accession: MI0008353) by polymerase chain reaction (PCR). The amplified products were sequenced and cloned into the pCAMBIA1300. The constructed plasmids were introduced into *Agrobacterium tumefaciens* strain GV3101. Subsequently, *A. tumefaciens* harboring the recombinant plasmids was transformed into tomato plants as reported previously by Sharma et al. (2009). To further screen the transgene, genomic DNA from primary transformants (T0) was used as the template to amplify the NPT-II gene (Porebski et al., 1997). The primers used for the construction of recombinant plasmids are listed in Supplementary Table 1.

### Virus-Induced Gene Silencing

To develop a virus-mediated gene silencing (VIGS) system for tomato, the virus vectors (pTRV1 and pTRV2) were applied in our experiments. The VIGS systems were carried out as reported previously by Liu et al. (2002). In the VIGS systems, cDNA fragment of phytoene desaturase gene (PDS) from tomato plants were inserted into the pTRV2. In addition, to construct the VIGS system for silencing other target genes, the corresponding cDNA fragments were inserted into the pTRV2, respectively. All the vectors including the pTRV1 and recombinant pTRV2 were introduced into *A. tumefaciens* strain GV3101. *A. tumefaciens* harboring the pTRV1 or pTRV2-derived plasmids were mixed in a volume ratio of 1:1. Subsequently, the mixture was infiltrated into 7-day-old tomato cotyledons. Finally, these treated plants were cultured for 3 weeks at 21°C. The primers used for the construction of TRV-based recombinant plasmids are supplemented in Supplementary Table 1.

### 3, 3′-Diaminobenzidine (DAB) and Trypan Blue Staining

For *in vivo* localization of H_2_O_2_, DAB staining was carried out to analyze the accumulation of H_2_O_2_ in leaves as described previously by Lee et al. (2002). Leaf tissues were immersed in the DAB staining solution (pH 3.0; 1 mg L^–1^ of DAB, 0.05% v/v Tween 20) and vacuum-filtrated for 5 min. Then, the stained leaves were incubated at room temperature in the dark for 4 h, followed by bleaching with destaining solution (acetic acid: ethanol: glycerol, 1:1:1) for 12 h. For detection of cell death, trypan blue staining was carried out according to the method (Jiang et al., 2018). Briefly, pathogen-infected leaves were incubated in 100 ml of staining solution (80 mg of trypan blue, 20 ml of sterile distilled water, 20 ml of lactic acid, 20 ml of glycerol and 20 ml of phenol) at room temperature in the dark for 30 min. Then, the stained leaves were bleached with destaining solution (acetic acid: ethanol, 1:3) for 6 h.

### Quantitative Real-Time (qPCR) Analysis

The transcription of lncRNAs, miRNAs and target genes was examined using qPCR analyses. The miRNA Universal SYBR qPCR Master Mix (Vazyme Biotech, Nanjing) was applied to conduct qPCR reactions of miRNAs. The qPCR reactions of lncRNAs and target genes were conducted using the SYBR Premix Ex TaqTM II kit (TaKaRa, Japan). All the reactions were conducted using an ABI 7500 PCR machine according to the method (Zhou et al., 2016; Meng et al., 2020). The *SlActin* gene was used as a control to normalize gene expression using the 2^–ΔΔ*CT*^ method. The primers used for qPCR reactions are supplemented in Supplementary Table 1.

### Statistical Analysis

The data were statistically analyzed by the IBM SPSS software. The data represented the means ± SD from three biological repeats. Significant difference between different experimental groups was analyzed using Tukey’s *post hoc* (^∗^*P* < 0.05, ^∗∗^*P* < 0.01; ns, not significant) or Duncan’s multiple range tests (different letters indicated statistically different at *P* < 0.05) for one way ANOVA.

## Results

### *Bacillus subtilis* SL18r Increased Tomato Resistance Against *B. cinerea* Infection

**FIGURE 1:**
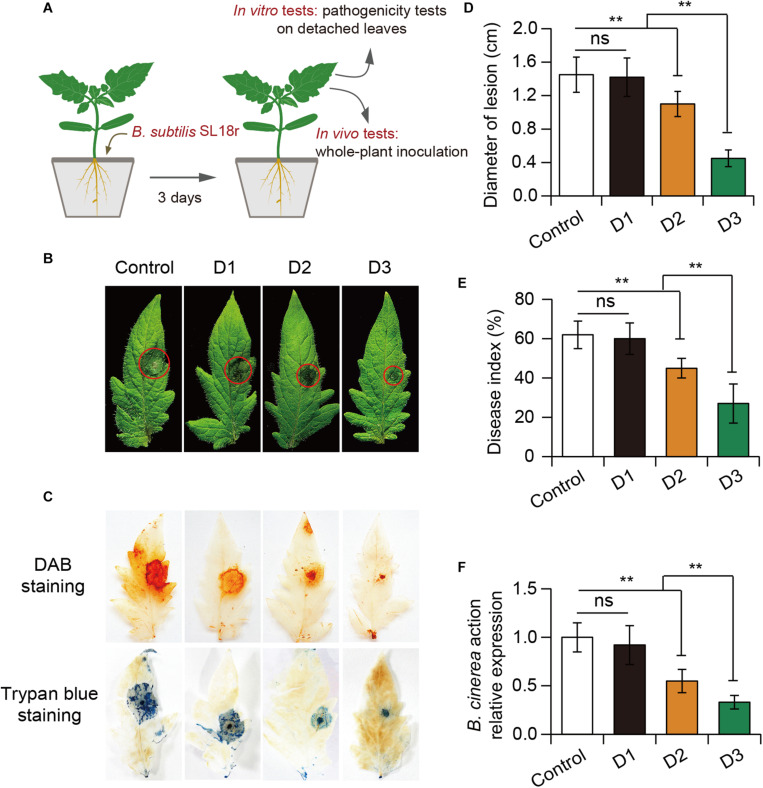
Soil inoculation with *Bacillus subtilis* SL18r increased tomato resistance against the invasion of *Botrytis cinerea*. **(A)** Seven-day-old tomato seedlings were initially cultivated in soil for 3 weeks and were then exposed to different doses of SL18r (D1: 4 × 10^6^, D2: 2 × 10^7^, and D3: 1 × 10^8^ CFU g^− 1^ of soil). After 3 days of culture, both the control and inoculated plants were used to perform *in vitro* and *in vivo* assays. **(B)** Phenotypes of detached leaves from both the control and inoculated plants at 5 dpi. Diaminobenzidine (DAB) and trypan blue staining **(C)** and lesion diameters **(D)** of the detached leaves. Moreover, in the whole-plant inoculation tests, the collected spore suspension of *B. cinerea* was applied to spray the leaves. Disease index **(E)** and *B. cinerea* actin gene expression **(F)** of the control and inoculated plants was detected at 5 dpi. Significant difference was analyzed using Tukey’s *post hoc* (**P < 0.01; ns, not significant).

To examine whether rhizo-inoculation with *B. subtilis* SL18r enhanced the ability of tomato plants to withstand the invasion of *B. cinerea*, pot experiments were conducted (Figure 1A). Seven-day-old tomato seedlings were firstly cultured in soil for 3 weeks and were then poured with cell suspension of SL18r at the different concentrations of 4 × 10^6^, 2 × 10^7^, and 1 × 10^8^ CFU g^–1^ of soil for 3 days, respectively, and tomato plants treated with the PBS solution were used as the control. Subsequently, 5 μl of 2 × 10^5^ spore ml^–1^ spore suspension of *B. cinerea* were inoculated on the detached leaves. As shown in Figure 1B, the inoculation with SL18r at the density of 4 × 10^6^ CFU g^–1^ soil could not suppress *B. cinerea* infection at 5 dpi. In contrast, higher inoculum dosage of SL18r at the density of 2 × 10^7^ or 1 × 10^8^ CFU g^–1^ soil distinctly reduced necrotic spots and lesion diameters on the detached leaves. DAB staining showed that the production of H_2_O_2_ were notably less in leaves of tomato plants treated with higher inoculum dosage of SL18r compared with the controls (Figure 1C). Trypan blue staining displayed more cell death in the control leaves compared with the inoculated leaves (Figure 1D). In the whole-plant inoculation tests, the control and inoculated plants were sprayed with 2 × 10^5^ spore ml^–1^ spore suspension of *B. cinerea* on the leaf surface for 5 days. As shown in Figure 1E, higher inoculum dosage of SL18r significantly lowered disease index in tomato plants compared with the controls. In line with the reduced disease occurrence, the SL18r-inoculated leaves displayed lower the expression of *BcActin* than the control leaves (Figure 1F). Our findings indicated that the increased disease resistance of plants conferred by SL18r was dependent on its inoculum dose.

### Identification of Differentially Expressed LncRNAs

**FIGURE 2:**
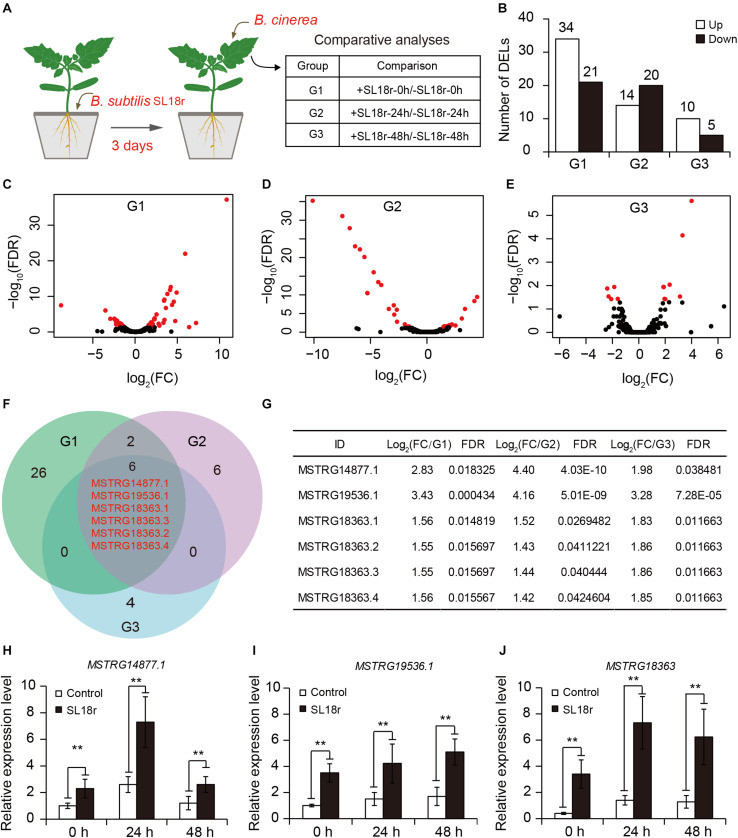
Identification of differentially expressed lncRNAs (DELs) between the control and SL18r-inoculated plants. **(A)** Seven-day-old tomato seedlings were initially cultivated in soil for 3 weeks and were then treated without (−SL18r) or with SL18r (+SL18r) at the final density of 1 × 10^8^ CFU g^− 1^ of soil for 3 days. Then, the leaves were sprayed with 2 × 10^5^ spore mL^− 1^ of spore suspension of *Botrytis cinerea* and were used to conduct RNA-Seq. **(B)** Number of DELs. **(C–E)** Volcano plot of DELs. Red points represented the DELs. **(F)** Venn diagram of upregulated DELs among different experimental groups. G1, +SL18r-0h/-SL18r-0h; G2, +SL18r-24h/-SL18r-24h; G3, +SL18r-48h/ -SL18r-48h. **(G)** The expression profiles of the shared DELs among different experiment groups. **(H–J)** qPCR analyses of the shared DELs. Significant difference was analyzed using Tukey’s *post hoc* (**P < 0.01).

To unravel the molecular responses of SL18r-inoculated plants to *B. cinerea* infection, RNA-Seq was conducted for comparative analyses of the lncRNA expression profiles between the control and inoculated plants. Tomato plants were inoculated with SL18r for 3 days. After 0, 24, and 48 h of pathogen infection, leaf samples were used for the extraction of total RNAs to perform RNA-Seq (Figure 2A). Compared with the controls, a total of 55, 34, and 15 lncRNAs as differentially expressed lncRNAs (DELs) in the inoculated plants were screened with a FDR-adjusted *P* < 0.05 and fold change >1.0 or <−1.0), including 34, 14, and 10 upregulated DELs, and 20, 21, and 5 downregulated DELs, respectively (Figures 2B–E and Supplementary Table 2). Among these DELs, a total of 6 upregulated DELs were commonly expressed in the inoculated plants at different pathogen-infected times (Figures 2F,G), which included MSTRG14877.1, MSTRG19536.1, and four alternatively spliced forms of MSTRG18363. In accordance to this, qPCR analyses showed that the SL18r-inoculated plants exhibited significantly higher transcription of MSTRG14877.1, MSTRG19536.1 and four variants of MSTRG18363 (which could not be distinguished by qPCR) as compared to the controls (Figures 2H–J).

### Activation of MSTRG18363 Expression Was Required for *B. subtilis* S18r-Induced Disease Resistance

**FIGURE 3:**
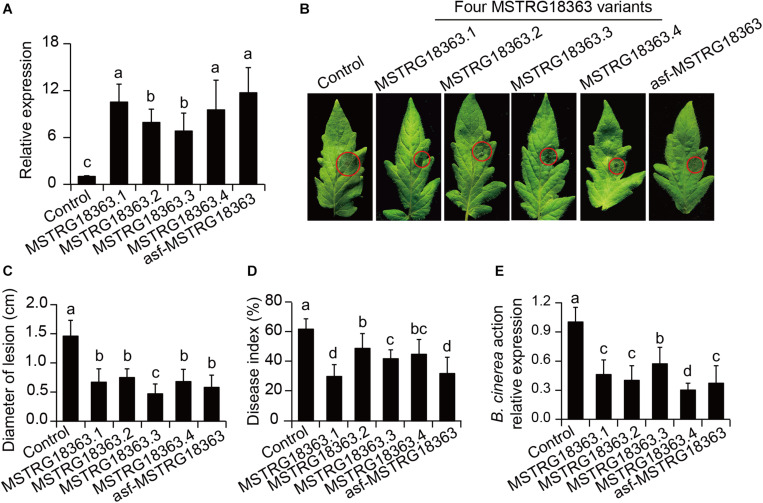
Overexpression of four MSTRG18363 variants and artificially synthesized form of MSTRG18363 (asf-MSTRG18363, consensus sequence of four MSTRG18363 variants) increased the ability of transgenic tomato plants to inhibit the infection of *Botrytis cinerea.* Four-week-old tomato plants were used to perform *in vitro* (pathogenicity tests on detached leaves) and *in vivo* (whole plant inoculation tests) assays. **(A)** qPCR analyses of MSTRG18363 expression in transgenic tomato plants. Phenotypes **(B)** and lesion diameters **(C)** of detached leaves from the MSTRG18363-overexpressing plants at 5 dpi. In the whole-plant inoculation tests, the leaves were sprayed with 2 × 10^5^ spore mL^− 1^ spore suspension of *B. cinerea*. Disease index **(D)** and the expression of *BcActin* **(E)** were analyzed at 5 dpi. Different letters represented significant difference using Duncan’s multiple range tests at *P* < 0.05 for one way ANOVA.

To explore the functions of MSTRG18363 in the responses of tomato leaves to the invasion of *B. cinerea*, the pCAMBIA1300-MSTRG18363 plasmids were transformed into tomato plants. Compared with the wide-type (WT) plants, four MSTRG18363 variants and an artificially synthesized form of MSTRG18363 (asf-MSTRG18363, consensus sequence of four MSTRG18363 variants; Supplementary Figure 1) were highly transcribed in transgenic plants (Figure 3A). In *in vitro* assays, after 5 days of *B. cinerea* infection, the WT plants displayed more serious disease symptoms with larger lesion diameters than all the transgenic plants (Figures 3B,C). In *in vivo* assays, these plants were infected by *B. cinerea* for 5 days. The SL18r-inoculated plants displayed significantly lower disease index than the controls (Figure 3D). Accordantly, the expression of *BcActin* was markedly less in the SL18r-inoculated leaves than the control leaves (Figure 3E). In addition, tomato plants overexpressed asf-MSTRG18363 also exhibited stronger resistance against *B. cinerea* compared with the WT plants (Figures 3B–E).

**FIGURE 4:**
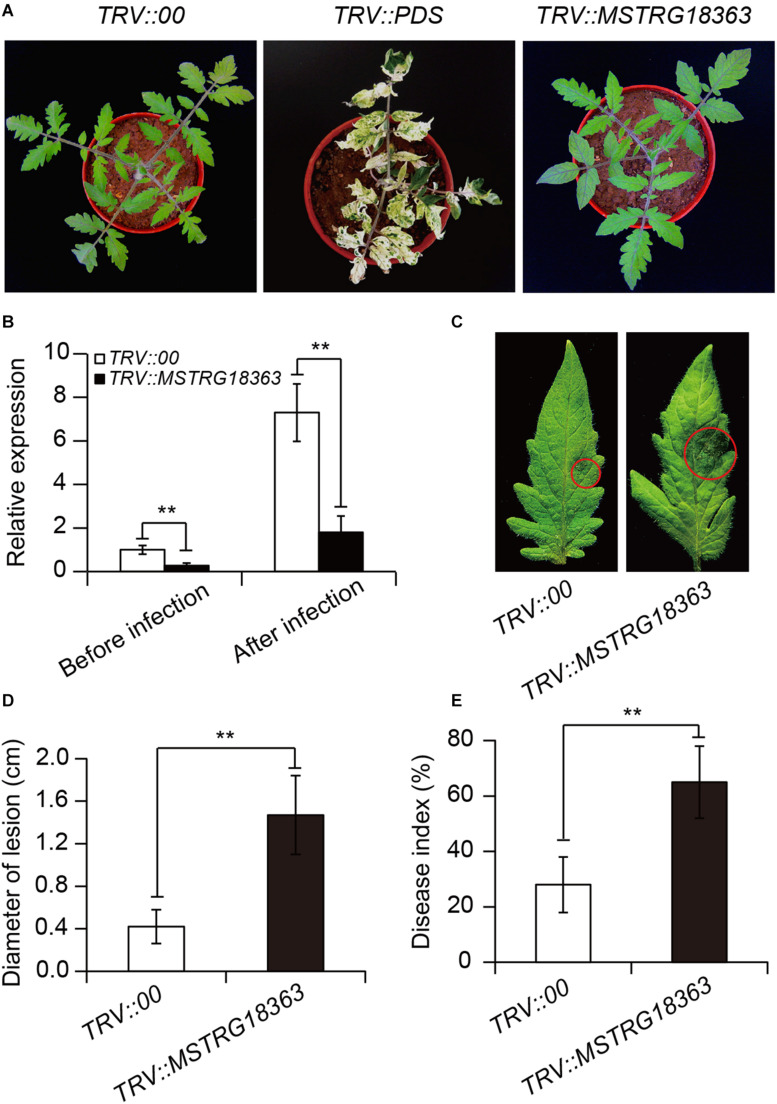
Silencing of MSTRG18363 largely weakened the SL18r-induced tomato resistance against the invasion of *Botrytis cinerea*. **(A)** Phenotype of tomato plants treated with *TRV::00*, *TRV::PDS* or *TRV::MSTRG18363* after 3 weeks of culture. **(B)** The expression of MSTRG18363 in both the leaves *TRV::00* and *TRV::MSTRG18363* tomato in the presence of SL18r before or after 5 days of *B. cinerea* infection. Phenotypes **(C)** and lesion diameters **(D)** of detached leaves from both the *TRV::00* and *TRV::MSTRG18363* plants at 5 dpi. In the whole-plant inoculation tests, the leaves were sprayed with 2 × 10^5^ spore mL^− 1^ of spore suspension of *B. cinerea*. **(E)** Disease index of the *TRV::00* and *TRV::MSTRG18363* plants was detected at 5 dpi. Significant difference was analyzed using Tukey’s *post hoc* (**P < 0.01).

Furthermore, the VIGS system was developed to silence the expression of MSTRG18363 for analyzing its roles in the SL18r-induced disease resistance. Compared with the *TRV::00* leaves, the tomato *PDS* gene was considerably suppressed in the *TRV::PDS* leaves, showing the photo-leaching symptoms (Figure 4A). Moreover, the cDNA fragment of MSTRG18363 was inserted into the TRV2 plasmids (*TRV::MSTRG18363*), and the *TRV::00* plants were used as the controls. After 3 weeks of VIGS treatment, these plants were subjected to treatment with SL18r for 3 days. The transcription levels of MSTRG18363 were observably lower in the *TRV::MSTRG18363* leaves than the *TRV::00* leaves (Figure 4B). However, the *TRV::00* plants displayed no phenotypic discrepancy with the *TRV::MSTRG18363* plants (Figure 4A). After 5 days of pathogen infection, the *TRV::00* leaves exhibited higher expression of MSTRG18363 than the *TRV::MSTRG18363* leaves (Figure 4B). At 5 dpi, detached leaves of the *TRV::MSTRG18363* plants exhibited more serious disease symptoms and larger lesion diameters than the *TRV::00* plants (Figures 4C,D). In *in vivo* assays, the *TRV::MSTRG18363* plants displayed higher disease index than the *TRV::00* plants (Figure 4E). Our data suggested that induction of MSTRG18363 expression by SL18r resulted in stronger resistance of tomato plants against *B. cinerea* infection.

### MSTRG18363 as a miRNA Decoy to Suppress the Expression of miR1918

**FIGURE 5:**
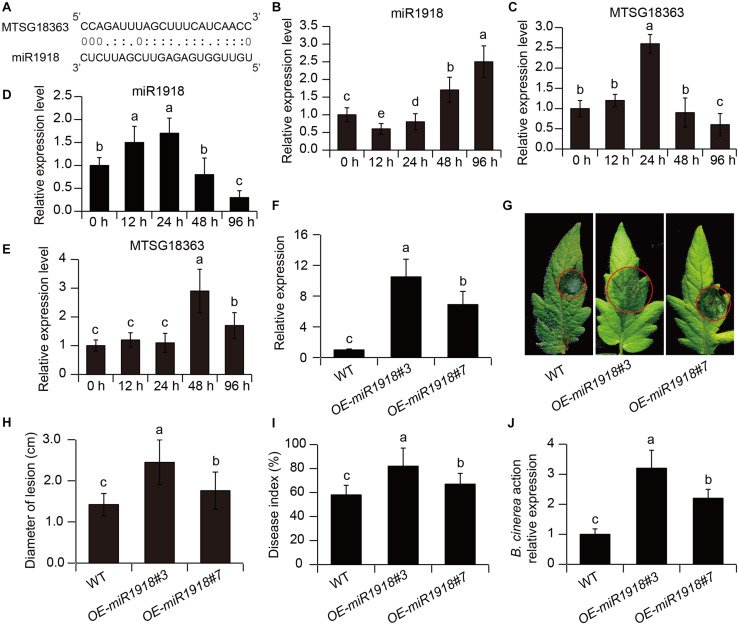
MSTRG18363 decoyed miR1918 in tomato plants. **(A)** Predicted base-pairing interactions between miR1918 and the binding site of MSTRG18363. The expression profiles of miR1918 and MSTRG18363 in both the control **(B,C)** and SL18r-inoculated **(D,E)** plants at different time points of pathogen infection. **(F)** The transcription of miR1918 in leaves of two independent miR1918-overexpressing tomato lines. **(G)** Phenotypes and **(H)** lesion diameters of detached leaves from the two independent transgenic lines at 5 dpi. Moreover, in the whole-plant inoculation tests, **(I)** disease index and **(J)** *Botrytis cinerea* actin gene expression of the control and transgenic lines was detected at 5 dpi. Different letters represented significant difference using Duncan’s multiple range tests at *P* < 0.05 for one way ANOVA.

As shown in Figure 5A, the binding site of miR1918 by MSTRG18363 was predicted using psRNATarget. To examine whether miR1918 and MSTRG18363 affected the responses of tomato plants to *B. cinerea* infection, their transcripts were quantified in the pathogen-infected leaves. The transcription levels of miR1918 were markedly increased in the non-inoculated (control) leaves at 48 h post infection (hpi), whereas the expression of MSTRG18363 was significantly reduced (Figures 5B,C). However, the opposite trends were observed for the expression of miR1918 and MSTRG18363 in the leaves of the SL18r-inoculated plants (Figures 5D,E). Moreover, the recombinant plasmid pCAMBIA1300-miR1918 was introduced into tomato plants. Compared with the wide-type (WT) plants, two independent miR1918-overexpressing lines (OE-miR1918#3 and #7) exhibited higher expression levels of miR1918 (Figure 5F). Detached leaves from both the WT and miR1918-overexpressing lines were subjected to the infection of *B. cinerea* for 5 days. The leaves from two miR1918-overexpressing lines displayed more serious disease symptoms than those of the controls (Figure 5G). Lesion diameters were also larger in the leaves of the miR1918-overexpressing lines than the controls (Figure 5H). In the whole-plant inoculation tests, disease index and abundance of *B. cinerea* was markedly higher in the two miR1918-overexpressing lines than the WT plants (Figures 5I,J).

**FIGURE 6:**
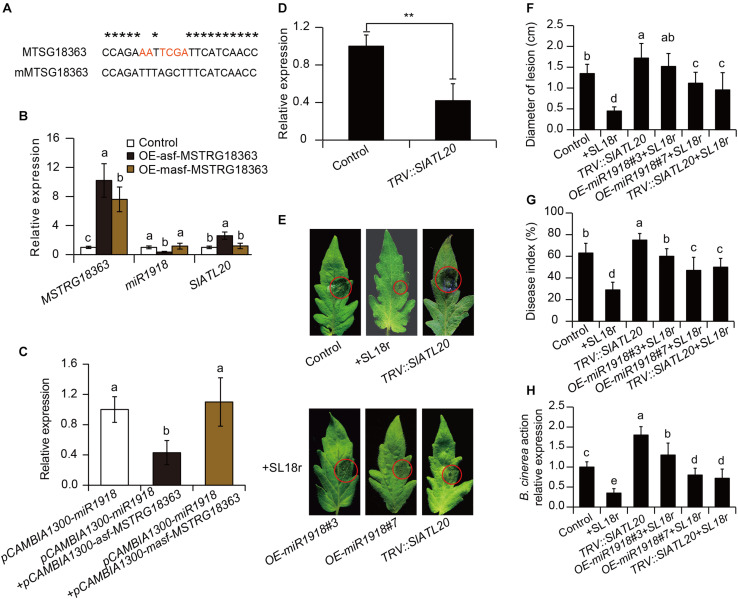
*Bacillus subtilis* SL18r-induced expression of MSTRG18363 functioned to mediate the *SlATL20* gene by decoying miR1918. **(A)** Predicted base-pairing interactions between miR1918 and the binding site of MSTRG18363 with the designed base mutation. Red letters indicated mutated bases. **(B)** qPCR analysis of MSTRG18363, miR1918 and *SlATL20* in the control, OE-asf-MSTRG18363, and OE-masf-MSTRG18363 tomato plants. **(C)** MSTRG18363 suppressed the expression of miR1918 in *Nicotiana* systems. The transcription of the miR1918 after the *Agrobacterium* strain harboring pCAMBIA1300-asf-MSTRG18363 and -masf- MSTRG18363 were introduced into the tobacco leaves that overexpressed miR1918. **(D)** The transcription of *SlATL20* in both the *TRV::00* and *TRV::SlATL20* tomato leaves. **(E)** Phenotypes of detached leaves from the *TRV::00*, SL18r-inoculated and *TRV::SlATL20* plants, and from the miR1918-overexpressing lines and *TRV::SlATL20* plants inoculated with SL18r at 5 dpi. **(F)** Lesion diameters of the detached leaves. Moreover, in the whole-plant inoculation tests, the leaves were sprayed with 2 × 10^5^ spore mL^− 1^ spore suspension of *Botrytis cinerea*. **(G)** Disease index and **(H)** *B. cinerea* actin gene expression of above the plants was detected at 5 dpi. Different letters represented significant difference using Duncan’s multiple range tests at *P* < 0.05 for one way ANOVA. Significant difference was analyzed using Tukey’s *post hoc* (**P < 0.01) or Duncan’s multiple range tests (different letters indicated statistically different at *P* < 0.05) for one way ANOVA.

To explore whether MSTRG18363 functioned by pairing with miR1918, base mutation was introduced into the paired sites of MSTRG18363 with miR1918 (mMSTRG18363) (Figure 6A). The transcription levels of miR1918 were investigated in the leaves of tomato plants that overexpressed MSTRG18363 by qPCR analyses. Overexpression of asf-MSTRG18363 reduced the transcription of miR1918 in tomato plants compared with the controls (Figure 6B). The regulation between MSTRG18363 and miR1918 was further analyzed by the transient expression system of *Nicotiana benthamiana*. The *Agrobacterium* strain harboring pCAMBIA1300-miR1918 was injected into leaf cells, which led to marked increases in the expression of miR1918 at 72 h compared with the controls. Then, the *Agrobacterium* strains carrying pCAMBIA1300-asf-MSTRG18363 and -masf-MSTRG18363 were injected into the leaves that overexpressed miR1918. As shown in Figure 6C, the expression of miR1918 was notably decreased after the introduction of asf-MSTRG18363, but was not found for masf-MSTRG18363. The expression of miR1918 was also less in the asf-MSTRG18363-overexpressing lines than the controls (Figure 6A). However, overexpression of masf-MSTRG18363 in tomato plants did not reduce the expression of miR1918 (Figure 6A). Furthermore, asf-MSTRG18363-overexpressing lines exhibited the increased transcription of the target gene *SlATL20* (Solyc01g095820 encoding a RING-H2 finger protein ATL20-like) of miR1918 (Luan et al., 2016). These results indicated that MSTRG18363 may function as a ceRNA for decoying miR1918 in tomato plants.

The VIGS system was further applied to explore the function of the target gene *SlATL20* of miR1918 in tomato plants. At 3 weeks after infiltration, the gene transcript abundance was greatly repressed in the leaves of *TRV::SlATL20* plants (Figure 6D). As shown in Figure 6E, more severe disease symptoms were observed in the *TRV::SlATL20* plants compared with the controls at 5 dpi (Figure 6E). The silenced plants had larger lesion diameters, higher disease index and more abundance of *B. cinerea* compared with the controls (Figures 6F–H). Furthermore, the SL18r-induced disease resistance was largely weakened in the *TRV::SlATL20* plants, as reflected by *in vivo* and *in vitro* assays of pathogen infection, which was similar to the observation for the miR1918-overexpressing lines (Figures 6E–H).

## Discussion

The molecular mechanisms underlying the PGPR-mediated ISR responses have recently been investigated extensively in many plant species such as tomato (Yan et al., 2002), *Arabidopsis* (Verhagen et al., 2004) and melon (García-Gutiérrez et al., 2013). The ISR processes involving several regulatory components have been well explored (Van Wees et al., 2008; Banks et al., 2012; Timmermann et al., 2019), but the roles of lncRNAs in the PGPR-induced ISR responses remain elusive so far. It is increasingly evidenced that lncRNAs can act as positive regulators of plant innate immunity: pathogen infection induces the expression of several miRNAs to suppress plant defense responses; meanwhile, a healthy plant is able to activate the lncRNA-mediated defense responses for decoying these negative regulators of defense systems (Jing et al., 2019; Hou et al., 2020; Jiang et al., 2020). In this study, beneficial rhizobacteria strain *B. subtilis* SL18r elevated the ability of tomato plants to inhibit the invasion of *B. cinerea*. Our results demonstrated that the SL18r-induced expression of MSTRG18363 conferred the increased host disease resistance via decoying miR1918, indicating that the PGPR-mediated ISR was closely associated with activation of lncRNA-mediated defense pathways.

Many studies have shown that LncRNAs can modulate the transcription of target genes *in cis* or *in trans* (Mercer and Mattick, 2013), and function as molecular scaffolds for recruiting the chromatin-modifying complexes (Fan et al., 2015; Holoch and Moazed, 2015). LncRNAs has also been reported to act as important regulators that bind to miRNA by the eTM sites in the sequences of lncRNAs and further inhibit the interactions between miRNA and its target mRNA, thereby enhancing the transcription of the target genes (Jing et al., 2019; Hou et al., 2020). IPS1 contains the eTM site to target the phosphate starvation-induced miR399 and thus increases the transcription of *PHO2* in *Arabidopsis* plants, thereby controlling the phosphate homeostasis (Ajmera et al., 2018). Similarly, PDIL1 can suppress the miR399-mediated *MtPHO2* degradation to control the P deficiency responses in *Medicago truncatula* (Wang et al., 2017). MLNC3.2 and MLNC4.6 can decoy the miR156a to inhibit the degradation of *SPL2-like* and *SPL33* genes in apple fruit, thereby regulating the light-induced anthocyanin biosynthesis (Yang et al., 2019). Overexpression of lncRNA23468 and lncRNA39026 in tomato plants reduces the expression of miR482b and miR168a, respectively (Jing et al., 2019; Hou et al., 2020), which contribute to the increased resistance of plants to *P. infestans*. However, it remains unclear whether the lncRNA-mediated pathways are involved in the PGPR-induced ISR responses. In this study, we examined which of the DELs between the control and SL18r-inoculated plants were likely involved in mediating the ISR responses. We further focused on the investigation of these DELs that potentially targeted miRNAs, which were involved in negative regulation of plant defense. Moreover, it was observed that four MSTRG18363 variants contained potential sites for binding to miR1918. The expression of MSTRG18363 was negatively related to the accumulation of miR1918 in the pathogen-infected leaves. Therefore, the interactions of MSTRG18363 with miR1918 may participate in the SL18r-mediated ISR processes in tomato plants.

It has previously been indicated that miR1918 negatively regulates plant defense systems in tomato plants (Luan et al., 2016). In tomato plants, miR1918 is present in target region of a putative RING finger gene *SlATL20* belonging to the zinc finger-coding family genes, which can mediate the growth and development, and stress adaption of plants (Zeng et al., 2006; Matthews et al., 2009; Luan et al., 2016). Several RING finger proteins are closely related to the mediation of plant ubiquitination, which is responsible for plant defense against abiotic and biotic stresses. For example, the transcription of *RFP1*, encoding a RING finger protein-coding gene from *Vitis pseudoreticulata*, is greatly induced in *V. pseudoreticulata* plants by the grapevine powdery mildew *Uncinula necator* (Van Wees et al., 2008). *Arabidopsis* plants overexpressing *VpRFP1* displays the increased resistance against *Arabidopsis* powdery mildew *Golovinomyces cichoracearum* and *Pseudomonas syringae* pv. *tomato* DC3000 (Yu et al., 2011). In *V. pseudoreticulata*, the RING finger protein gene *EIRP1* has been reported to regulate plant defense responses via promoting the proteolysis of VpWRKY11 (Yu et al., 2013). Furthermore, overexpression of *CaRING1* encoding a putative E3 ubiquitin ligase markedly enhances the resistance of *Arabidopsis* plant to *P. syringae* pv *tomato* and *Hyaloperonospora arabidopsidis* (Lee et al., 2011). Our findings revealed that the expression of miR1918 was negatively correlated with the expression of *SlATL20* and that there were more serious disease symptoms in the miR1918-overexpressing plants, indicating that miR1918 was involved in the silencing of *SlATL20*. Furthermore, silencing of *SlATL20* resulted in the increased susceptibility of tomato plants to *B. cinerea* infection. Therefore, we speculated that MSTRG18363 activated the ISR responses by regulation of miR1918-mediated plant ubiquitination. To confirm this hypothesis, the impacts of MSTRG18363-overexpression on disease resistance were assessed. Overexpression of MSTRG18363 reduced the expression of miR1918 and enhanced disease resistance in transgenic tomato plants, while silencing of MSTRG18363 increased the transcription levels of miR1918 and reduced disease resistance in the SL18r-inoculated plants. These observations were in concert with our conclusion that MSTRG18363 were positively involved in priming plants from *B. cinerea* infection via decoying miR1918.

## Conclusion

**FIGURE 7:**
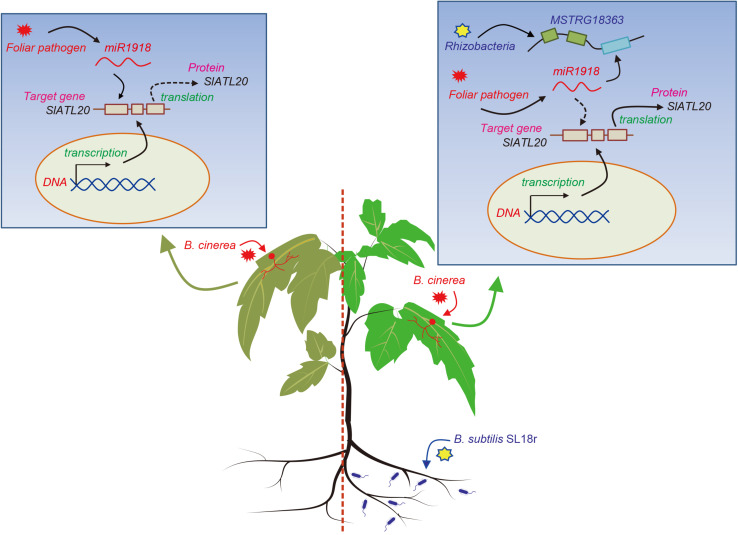
A proposed model was illustrated for *Bacillus subtilis* SL18r-induced ISR. When tomato plants were infected by the foliar pathogen *Botrytis cinerea*, the expression of miR1918 was considerably increased. However, high-level transcription of miR1918 suppressed the expression of the target gene *SlATL20* and further negatively regulated plant immune systems. Upon exposure to *B. subtilis* SL18r, the expression of MSTRG18363 was greatly stimulated in the leaves of tomato plants. Subsequently, the *B. cinerea*-initiated miR1918 was quickly decoyed by MSTRG18363, which prevented the miR1918-mediated inhibition of *SlATL20* transcripts. This contributed to the increased resistance of SL18r-inoculated plants against *B. cinerea* infection.

In summary, previous studies exploring the mechanisms of PGPR-induced ISR have been focused on the functions of protein regulators, miRNAs, JA-, and SA-signaling pathways, yet the involvement of lncRNAs in the rhizobacteria-primed ISR processes has not been investigated so far. Our data demonstrated that beneficial rhizobacteria strain *B. subtilis* SL18r was able to increase the resistance of tomato plants against the invasion of *B. cinerea* via the MSTRG18363-mediated decoy of miR1918, thereby contributing to efficient activation of ISR. As shown in Figure 7, a new model was proposed for illustrating the mechanisms of the SL18r-mediated disease resistance, in which pathogen infection induced a marked increase in miR1918 transcripts and thus inhibited the expression of *SIATL20* (the target gene of miR1918), leading to the reduced disease resistance. Upon infection in a primed state, the expression of the pathogen-initiated miR1918 was quickly quenched. The rhizobacteria-induced host resistance against foliar pathogens was in such an effective manner that did not require for activating a series of pathogenesis-related proteins and best fitted plant growth.

## Data Availability Statement

The datasets presented in this study can be found in online repositories. The names of the repository/repositories and accession number(s) can be found in the article/Supplementary Material.

## Author Contributions

CY and CZ conceptualized the research, responsible for the funding acquisition, and did the supervision. JZ and NQ conducted the investigation and formal analysis. CZ and JZ conducted the experiments. CZ, JZ, and NQ analyzed the results. CY, JG, and CZ wrote the original draft. JZ, JG, and NQ reviewed and edited the manuscript. All authors contributed to the article and approved the submitted version.

## Conflict of Interest

The authors declare that the research was conducted in the absence of any commercial or financial relationships that could be construed as a potential conflict of interest.

## Supplementary Material

Supplementary Figure 1 Sequence analyses of four MSTRG18363 variants.

Supplementary Figure 2 Consensus sequence of four MSTRG18363 variants (*asf-MSTRG18363*).

Supplementary Figure 3 Mutated sequence of asf-MSTRG18363 (*masf-MSTRG18363*).

Supplementary Table 1 Primers used in this study.

Supplementary Table 2 Up-regulated and down-regulated differentially expressed lncRNAs (DELs) in the SL18r-inoculated plants compared with the non-inoculated (control) plants.
