# Supplementary material for: Bacillus subtilis SL18r Induces Tomato Resistance Against Botrytis cinerea, Involving Activation of Long Non-coding RNA, MSTRG18363, to Decoy miR1918
Source: Front Plant Sci. 2021 Feb 3;11:634819. doi: 10.3389/fpls.2020.634819 (PMC7887324; doi:10.3389/fpls.2020.634819)
Supplement: Supplementary Figure 1 — Sequence analyses of four MSTRG18363 variants. [file Data_Sheet_1.PDF]

Figure S1

```
MSTRG.18363.1   GCAGAAAATAACATGTATTACTATATGAAGATTGGGTTGTAACATAAGCAACCACCAATGTTCATTACAAGACTCGACAA    80
MSTRG.18363.2   GCAGAAAATAACATGTATTACTATATGAAGATTGGGTTGTAACATAAGCAACCACCAATGTTCATTACAAGACTCGACAA    80
MSTRG.18363.3   GCAGAAAATAACATGTATTACTATATGAAGATTGGGTTGTAACATAAGCAACCACCAATGTTCATTACAAGACTCGACAA    80
MSTRG.18363.4   ...........CATGTATTACTATATGAAGATTGGGTTGTAACATAAGCAACCACCAATGTTCATTACAAGACTCGACAA    69
Consensus                  catgtattactatatgaagattgggttgtaacataagcaaccaccaatgttcattacaagactcgacaa

MSTRG.18363.1   TATATAAGTACTCCAAATTACAAAAGAAAAGAGACAACATATATATATATATATATATATATATATATATAT....AATG   156
MSTRG.18363.2   TATATAAGTACTCCAAATTACAAAAGAAAAGAGACAACATATATATATATATATATATATATATATATATATAT..AATG   158
MSTRG.18363.3   TATATAAGTACTCCAAATTACAAAAGAAAAGAGACAACATATATATATATATATATATATATATATATATATATATAATG   160
MSTRG.18363.4   TATATAAGTACTCCAAATTACAAAAGAAAAGAGACAACATATATATATATATATATATATATATATATATAT....AATG   145
Consensus       tatataagtactccaaattacaaaagaaaagagacaacatatatatatatatatatatatatatatatatat    aatg

MSTRG.18363.1   ATGGATCAAACTCGATAGATAAATCCATCTTATTCTTCATCTCCACAAGCCAGATTTAGCTTTCATCAACCTTACTGGAC   236
MSTRG.18363.2   ATGGATCAAACTCGATAGATAAATCCATCTTATTCTTCATCTCCACAAGCCAGATTTAGCTTTCATCAACCTTACTGGAC   238
MSTRG.18363.3   ATGGATCAAACTCGATAGATAAATCCATCTTATTCTTCATCTCCACAAGCCAGATTTAGCTTTCATCAACCTTACTGGAC   240
MSTRG.18363.4   ATGGATCAAACTCGATAGATAAATCCATCTTATTCTTCATCTCCACAAGCCAGATTTAGCTTTCATCAACCTTACTGGAC   225
Consensus       atggatcaaactcgatagataaatccatcttattcttcatctccacaagccagatttagctttcatcaaccttactggac

MSTRG.18363.1   CCTGCAATGCAATATTAAATCAAC..ATTAAATATATAGTATGCGCGTACACACATATATATCGTTTTGTAACATATAAG   314
MSTRG.18363.2   CCTGCAGTTGAGCAATCAGTGGAAGGGCTGATCTTGTAAGGAATGTTGACTC.CACAAACTCCAGGGAGCCCAGCGGCTT   317
MSTRG.18363.3   CCTGCAATGCAAT...................................................................   253
MSTRG.18363.4   CCTGCAGTTGAGCAATCAGTGGAAGGGCTGATCTTGTAAGGAATGTTGACTC.CAC........................   280
Consensus       cctgca t  a

MSTRG.18363.1   TTTAATCAATAGTCAGACATATATAAATTACAAATAAAAGAGATATTATACTGTCAATTGAATAAGTATAATCACCGTAC   394
MSTRG.18363.2   TTCCTGTATCAAT...................................................................   330
MSTRG.18363.3   ................................................................................   253
MSTRG.18363.4   ................................................................................   280
Consensus

MSTRG.18363.1   GTTGAGCAATCAGTGGAAGGGCTGATCTTGTAAGGAATGTTGACTCCACAAACTCCAGGGAGCCCAGCGGCTTTTCCTGT   474
MSTRG.18363.2   ................................................................................   330
MSTRG.18363.3   ................................................................................   253
MSTRG.18363.4   ................................................................................   280
Consensus

MSTRG.18363.1   ATCAA                                                                              479
MSTRG.18363.2   .....                                                                              330
MSTRG.18363.3   .....                                                                              253
MSTRG.18363.4   .....                                                                              280
Consensus
```

Figure S2

CATGTATTACTATATGAAGATTGGGTTGTAACATAAGCAACCACCAATG
TTCATTACAAGACTCGACAATATATAAGTACTCCAAATTACAAAAGAA
AAGAGACAACATATATATATATATATATATATATATATATATATAATGATGG
ATCAAACTCGATAGATAAATCCATCTTATTCTTCATCTCCACAAG**CCAG**
ATTTAGCTTTCATCAACCTTACTGGACCCTGCA

Figure S3

CATGTATTACTATATGAAGATTGGGTTGTAACATAAGCAACCACCAATG
TTCATTACAAGACTCGACAATATATAAGTACTCCAAATTACAAAAGAA
AAGAGACAACATATATATATATATATATATATATATATATATATATAATGATGG
ATCAAACTCGATAGATAAATCCATCTTATTCTTCATCTCCACAAG**CCAG**
AAA**T**TCGA**TTCATCAACC**TTACTGGACCCTGCA
